# Extraction of Teeth, by Dr. Cushman

**Published:** 1851-10

**Authors:** 


Extraction of Teeth by Dr. Cushman.—We should be glad to get many such articles presenting the difficulties in practice and the method of overcoming them. We may differ with the Doctor in the use of the Turnkey to the tooth described, for we generally use for such cases a root forcep, the points sharpened so as to allow of being forced into the socket. If we find the shell breaking, we urge on the forcep, and by a continuous shaking or rotatory motion, generally loosen the tooth. Yet teeth will often break, and, with the Doctor, we have no confidence in the assertion of those who say they have invariable success.

